# Nitrodiazene oxides: a unique nitrogen- and oxygen-containing functional group

**DOI:** 10.1098/rsos.231935

**Published:** 2024-03-28

**Authors:** Nicholas F. Scherschel, Davin G. Piercey

**Affiliations:** ^1^ School of Materials Engineering, Purdue University, West Lafayette, IN 47906, USA; ^2^ Purdue Energetics Research Center, Purdue University, West Lafayette, IN 47906, USA; ^3^ School of Mechanical Engineering, Purdue University, 585 Purdue Mall, West Lafayette, IN 47907, USA

**Keywords:** nitrodiazene oxides, nitrogen/oxygen functional groups

## Abstract

Nitrogen-/oxygen-containing functional groups (N/O groups) may be found in a wide variety of areas such as agriculture, drug design and energetic materials. Exploring the chemistry and synthesis of N/O groups is desirable as compounds containing their functionality may prove to be invaluable in a variety of fields. A unique N/O functional group which may offer additional insight into the design of high-heteroatom content systems is the 2-nitrodiazene-1-*N*-oxide group (NDO group). While unique on their own, NDOs combine the well-known azoxy (N(O)=N) and nitro (–NO_2_) groups into a single unique N/O functional group. Although NDOs may offer superior densities and enthalpies of formations relative to their nitro counterparts, NDOs have been significantly less investigated than their nitro-bearing counterparts. This work will discuss NDOs in chemical literature from their initial discovery to modern synthesis techniques, energetic properties and chemical stability.

## 1. Introduction

Nitrogen-/oxygen-containing functional groups (N/O groups) have a wide range of applications, which include, but are not limited to, medicine [1], pesticides [2] and energetic materials [3,4]. Despite possessing a wide range of applicability, there are relatively few unique N/O functional groups in the literature. Important N/O groups include the nitro, nitroso, hydroxylamine and the azoxy group. Nitro groups, in addition to being ubiquitous in the field of energetic materials [5,6], are used in drug design [1] and pesticides [2]. Nitroso groups are not widely used as they are susceptible to hydrolysis [7–9] and are suspected to be extremely carcinogenic to humans and animals [10,11]. However, nitroso groups do play important roles as synthetic intermediates in chemical synthesis [12]. Hydroxylamines are found in a variety of organisms [13,14], are thought to play an important role in the nitrogen cycle [15] and play important roles as intermediates in the synthesis of energetic materials. Azoxy groups have been shown to allow for liquid crystal functionality [16], are known to undergo photochemical rearrangements [17] and may improve energetic performance with their *N*-oxide (superior density, oxygen balance, detonation velocities and detonation pressures) [18,19].

Additionally, N/O groups also play important roles in the synthesis of high-heteroatom content heterocycles. For example, a variety of exotic compounds like 1,2,3,4-tetrazine-1,3-dioxides [20], several pentazoles [21] and 1,3-bis(nitroimide)-1,2,3-triazolate [22] are known in the literature and are unusually stable given their high-heteroatom (especially nitrogen) content. An excellent way to understand the stability of high-heteroatom content structures such as these is by studying the stability of compounds which contain fundamental N/O functional groups.

For energetic materials, N/O-containing functional groups are invaluable as they serve to increase the enthalpy of formation and increase the oxygen balance of the material. In the synthesis of energetic materials, there are three major design motifs which are the basis for performance: incorporation of fuel and oxidizer in the same material, increasing the enthalpy of formation by introducing ring/cage strain, and incorporation of many N–N single and double bonds. N/O-containing groups serve to provide the oxidizer in the form of oxygen (nitro, hydroxylamine and azoxy) and increase the enthalpy of formation by introducing N–N bonding (azoxy). A major issue for most energetic materials used today, like 1,3,5-trinitroperhydro-1,3,5-triazine (RDX) and 2,4,6-trinitrotoluene (TNT), is that they do not carry sufficient oxidizer in the molecule to oxidize all carbon and hydrogen in the molecule to CO, CO_2_, H_2_O and other decomposition products upon detonation. Incorporating additional N/O groups into energetic materials may serve to address the oxygen balance shortcoming by simultaneously reducing the carbon content and increasing the oxygen content of materials.

A unique N/O group is the 2-nitrodiazene-1-*N*-oxide group (NDO group) which combines the well-known functional groups of azoxy (N(O)=N) and nitro (–NO_2_) into a unique combination of the two. While there are a plethora of examples of axozy- and nitro-containing compounds, there are fewer than three dozen molecules to date which contain the NDO motif. NDOs are a highly unique and relatively underexplored functional group which possess high enthalpies of formation and high oxygen content. Accordingly, NDOs should be of interest to a variety of disciplines as they serve to address fundamental problems in energetic materials design and further the understanding of molecular stability in highly unique N/O group systems [22,23].

## 2. Nitrodiazene oxides

### 2.1. Introduction/history

While the first spectroscopic analyses and published syntheses of NDOs were reported beginning in 1994 and 1996, respectively, the earliest mention of NDOs dates back originally to 1991 in a publication on 1,2,3,4-tetrazine-1,3-dioxides by Tartakovsky *et al.* [24]. However, the article only mentions NDOs passingly and directs readers to view future publications for additional details. The following year in 1992, some computational studies on the predicted enthalpy of formation of NDOs were reported in the Proceedings of the Eighteenth International Pyrotechnics Seminar by Marchenko [25]. Marchenko’s findings indicated NDOs could provide superior enthalpies of formation compared with compounds which possess a nitro group in an equivalent position as NDO for use as energetic materials. While the time of the first synthesis is unclear, it appears that the first attention was given to the NDO moiety by a variety of Soviet, and then later Russian, groups in the 1980s and 1990s.

### 2.2. Spectroscopic investigations of NDOs

**Figure 1.**
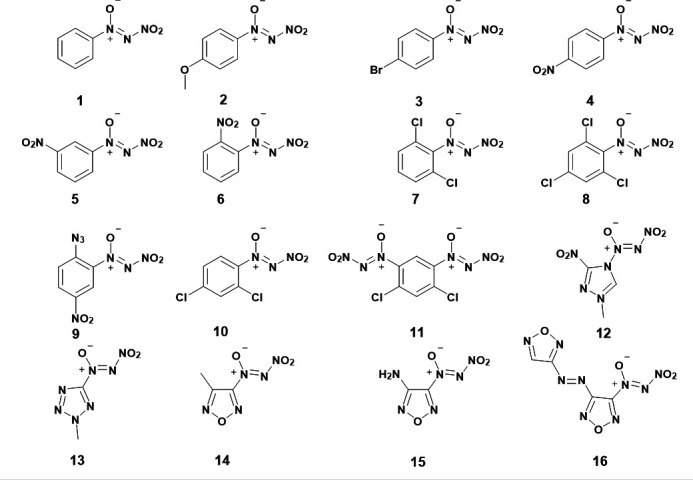
Reported NDOs synthesized by Tartakovsky in 1994 after his seminal work [26,27].

The first spectroscopic investigations with NDOs were reported in 1994 by Tartakovsky *et al.* [26,27]. In the seminal paper on NDOs, specific IR absorption bands of select NDOs were reported [26], but few months later a more complete list of reported NDOs was presented in a second publication [27]. NDOs reported in the first and second spectroscopic studies may be seen in figure 1. Characterization of the NDO moiety was possible by ^15^N labelling of the 1-*N*-oxide nitrogen in the NDO group. This work identified the unique IR and Raman spectra in the NDO group across many different backbones; however, experimental conditions for the synthesis of NDOs were not reported until 1996.

### 2.3. Synthesis of nitrodiazene oxides

**Scheme 1:.**
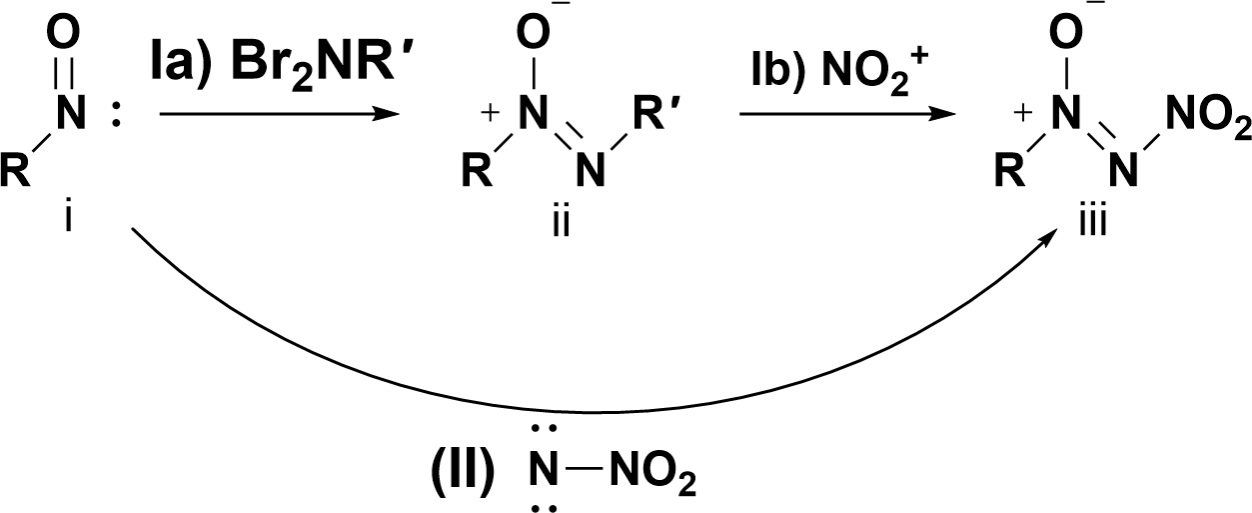
Two predominant synthetic motifs of synthesizing NDOs as described by Tartakovsky [28]. R is a heterocyclic or straight-chain carbon, R´ is commonly tBu or Ac and the counterion for NO_2_^+^ is BF_4_^–^ or SiF_6_^2–^.

The first reported synthetic procedure for synthesizing NDOs was first described by Tartakovsky in 1995 in a conference publication [28]. In his publication, he briefly, without full experimental details, discusses two ways that his group has been able to synthesize NDOs. (i) The first method is chemical in nature and relies upon the reaction of a nitroso compound with *N*, *N*-dibromo-tert-butylamine (t-BuNBr_2_) to yield an isolatable intermediate (ii) bearing a tert-butyl-azoxy functionality (scheme 1). The reaction of a nitroso with dibromamines was initially described by Zawalski & Kovacic in 1979 [12]. Next, the tert-butyl-azoxy intermediate may then be reacted with an NO_2_^+^ source to yield the NDO. Alternatively, a one-step electrochemical coupling of a nitronitrene, generated from nitramide, with a nitroso compound is said to be possible (scheme 1(II)); however, no further mention of this method is made by Tartakovsky [28]. Although this article is the first to describe the synthesis of NDOs and was published nearly 30 years ago, the synthetic steps for synthesizing NDOs remain essentially unchanged to the present.

**Scheme 2:.**
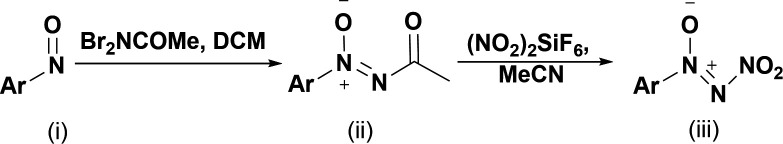
Synthetic procedure for NDOs on aryl groups as reported by Tartakovsky *et al.* in 1996 [29].

**Figure 2.**
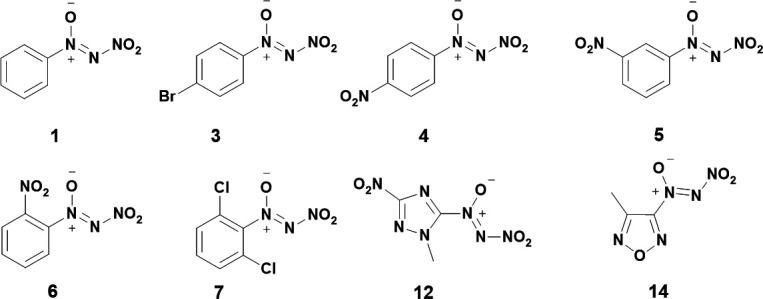
Structures reported for NDOs from Tartakovsky *et al.*’s 1996 work [29].

The first synthesis of NDOs was first fully formally published in 1996 by Tartakovsky *et al.* [29]. In this work, a nitroso group (scheme 2(i)) is reacted with N,N-dibromoacetamide (Br_2_NCOMe) to yield an intermediate azoxy (scheme 2(ii)), which is then purified and reacted with (NO_2_)_2_SiF_6_ in acetonitrile to yield the corresponding NDO [29]. The general scheme for this route is shown in scheme 2. Aryl groups which successfully underwent the reaction as outlined in scheme 2 were nitrosobenzene, *para*-bromo-nitrosobenzene, *ortho*-, *meta*- and *para*-nitro-nitrosobenzene and 2,6-dichloro-nitrosobenzene to yield the previously described compounds of **1**, **3–7**, **12** and **14**. Yields for isolated NDOs ranged from 68% to 91%. Tartakovsky also reports the syntheses of two additional NDOs on furazan and triazole backbones, the structures are shown in figure 2. For **12** and **14**, the syntheses remain identical, except the NO_2_^+^ source is NO_2_BF_4_ instead of (NO_2_)_2_SiF_6_. According to Tartakovsky, NO_2_BF_4_ was not used for aryl NDOs as the BF_4_^–^ anion degrades to BF_3_, which resulted in poor yields of aryl NDOs by side reactions with HF.

**Figure 3.**
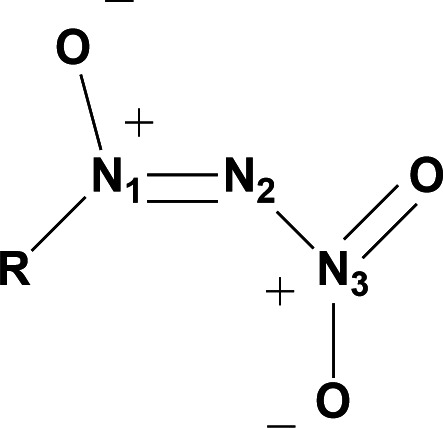
General structure of NDO structural motif with numbering. R is 1-(4-bromophenyl) or 1-(2,6-dichlorophenyl). Selected bond length of (**3**) N(3)–N(2) 1.48 Å and (**7**) N(3)–N(2) 1.475 Å.

Decomposition data were gathered for all NDOs and generally were found to range from 85 to 110°C. Additionally, crystal structures were provided for 1-(4-bromophenyl)-2-nitrodiazene 1-oxide (**3**) and 1-(2,6-dichlorophenyl)-2-nitrodiazene 1-oxide (**7**). Of particular interest is the abnormally long N(3)–N(2) bond length of approximately 1.48 Å (figure 3), which is longer than the average N–N bond length of 1.40 Å for RDX [30] and slightly longer than the average N–N bond length of 1.47 Å [31]. This unusually long N–NO_2_ bond may be explained by the terminal nitro group lying essentially entirely out of plane at −99.1° for **3** and −97.1° for **7** [29]. Having the terminal nitro group out of plane relative to the rest of the NDO group means that sharing of π electrons is restricted, thus weakening the bond and simultaneously increasing the N–NO_2_ bond length relative to other N–NO_2_ bonds.

**Scheme 3:.**
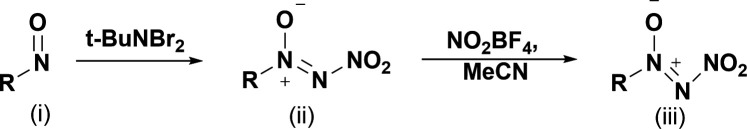
General scheme for synthesis of NDOs. For **17** and **18,** R is a 1,2,5-oxadiazole [33].

**Figure 4.**
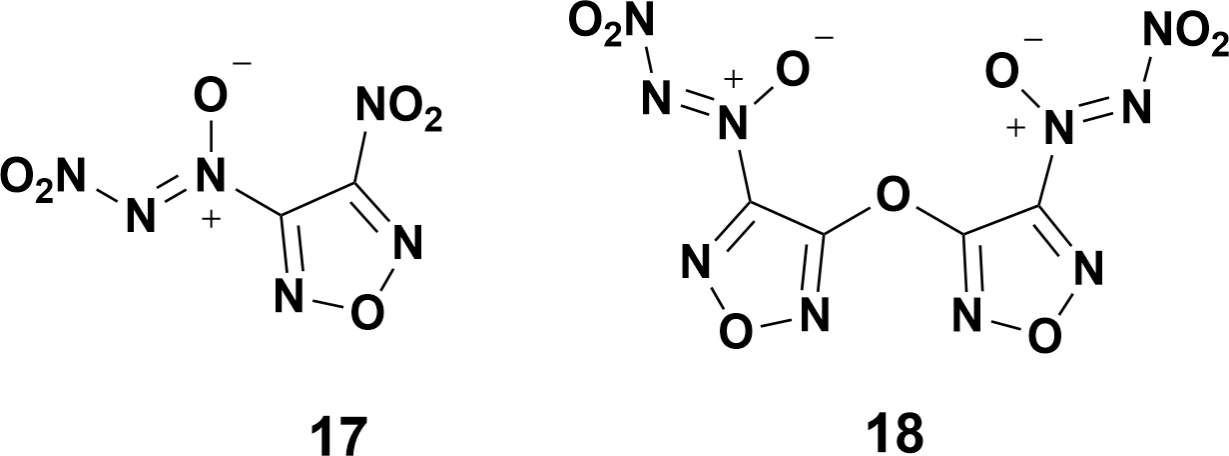
Structures of (Z)-2-nitro-1-(4-nitro-1,2,5-oxadiazol-3-yl)-diazene-1-oxide **17** and (1Z,1´Z)-1,1´-(oxybis(1,2,5-oxadiazole-4,3-diyl))-bis-(2-nitrodiazene-1-oxide) **18**.

**Figure 5.**
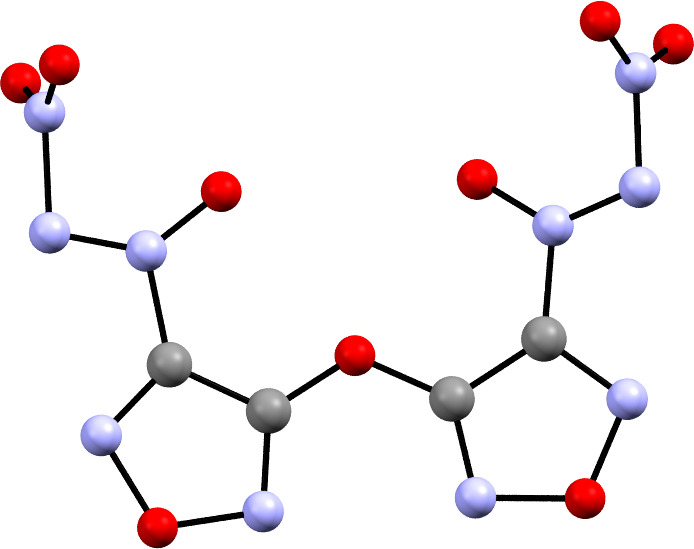
Crystal structure of **18** reproduced from Ioffe *et al.*’s 1998 NDO paper [33].

The next paper on NDOs came the following year by Tartakovsky *et al.*, which reports on an improved synthesis compared with his previous study [32]. The new synthesis found that reacting a nitroso compound (scheme 3(i)) with t-BuNBr_2_ to yield a tert-butyl azoxy group (scheme 3(ii)) tended to provide the most easily substituted intermediate which yields the final NDO (scheme 3(iii)) after reaction with NO_2_BF_4_ (scheme 3) [32]. The next major paper on the synthesis of new NDOs was published in 1998 by Ioffe *et al.*, which capitalized upon the improved synthesis [32]. In the 1998 paper, Ioffe was able to synthesize two furazans (figure 4) by similar methodology to the landmark 1996 NDO synthesis paper with slight modifications to the original synthesis (scheme 3). In this case, the slight modification originates with the azoxy addition. The t-BuNBr_2_ is reacted with a nitroso by the Kovacic method [12] to yield the intermediate azoxy, which is then nitrated with NO_2_BF_4_ to yield the final NDO. A summary is provided in scheme 3. Ioffe’s 1998 paper features a much more energetic backbone in 3-nitro-1,2,5-oxadiazole in addition to an optimized synthesis route compared with the original 1996 paper by Tartakovsky. Notably, Ioffe was able to synthesize a unique bridged furazan ether system with two NDO groups in **18**. Ioffe also included a crystal structure of **18** (figure 5), which boasts an N–NO_2_ bond length of 1.503 Å, even longer than identified in **3** and **7**. Little alteration to the procedure for the synthesis of NDOs has occurred since this report by Ioffe.

**Scheme 4:.**
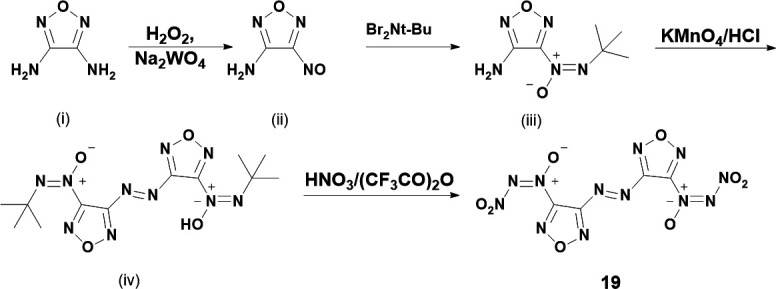
Synthetic procedure for synthesis of **19**. Scheme is reproduced from work by Fan *et al.* [34].

**Scheme 5:.**
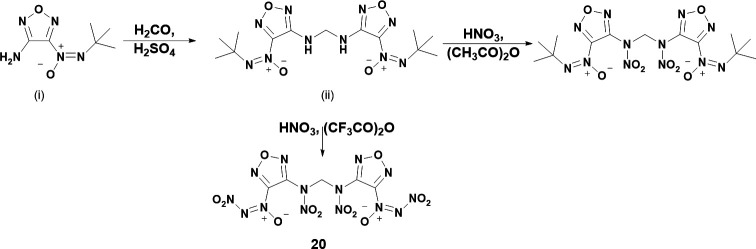
Synthetic procedure for **20**. Scheme is reproduced from work by Wang *et al.* [35].

The next reported synthesis on NDOs arrived over a decade later in 2013 by Fan *et al.* [34]. Fan reports synthesis of **19** starting with 3,4-diamino furazan (DAF, scheme 4(i)). DAF is reacted with hydrogen peroxide and sodium tungstate to yield 4-nitroso-1,2,5-oxadiazole-3-amine (scheme 4(ii)), which is then reacted with t-BuNBr_2_ to yield the intermediate tert-butyl-azoxy (scheme 4(iii)) by the aforementioned Kovacic method [12]. Next, the intermediate azoxy is reacted with potassium permanganate and hydrochloric acid to azo-couple the azoxy furazans (scheme 4(iv)). Finally, the azo-coupled intermediate is then nitrated with white fuming nitric acid (WFNA) and trifluoroacetic anhydride to yield the final NDO **19** (scheme 5).

The following year, Wang *et al.* reported another synthesis of a NDO again involving DAF as the starting material [35]. In Wang’s synthesis of **20**, 3-amino-4-tert-butyl-azoxy-1,2,5-oxadiazole (scheme 5(i)) is reacted with formaldehyde in the presence of sulphuric acid to yield the methylene intermediate (scheme 5(ii)). The intermediate is then reacted with WFNA in the presence of acetic anhydride and trifluoroacetic anhydride to yield nitramines with the former and nitramines with NDOs on **20**. A summary of the reaction for **20** may be seen in scheme 5. It seems that trifluoroacetic anhydride is required for loss of the tert-butyl group as acetic anhydride/WFNA does not yield loss of the tert-butyl group in this instance.

**Scheme 6:.**
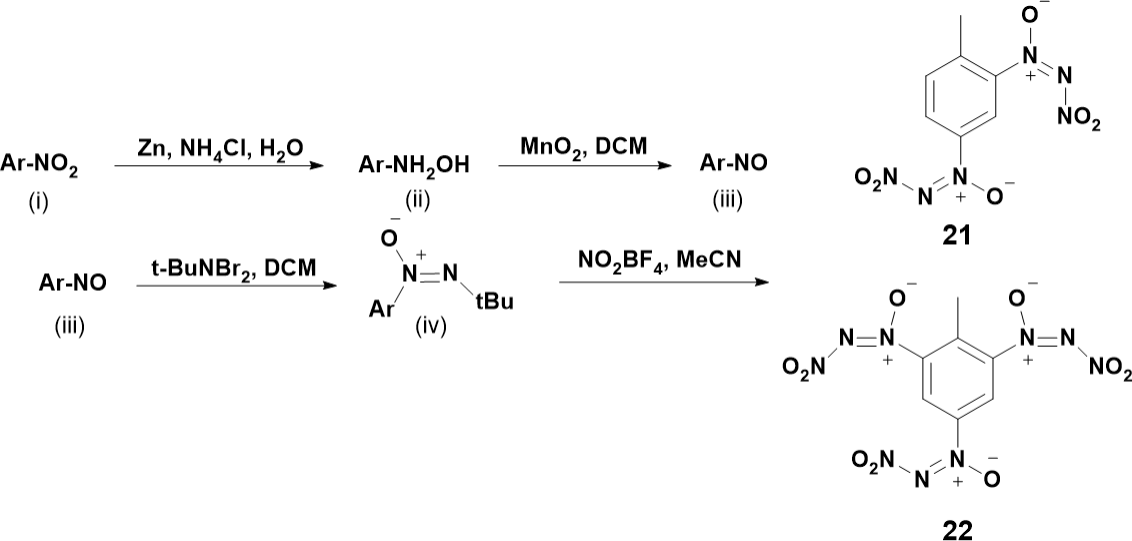
Synthetic procedure for **21** and **22** by Pang *et al*. where Ar is DNT or TNT [36].

In 2014, Pang and co-workers synthesized analogous energetics to that of dinitrotoluene (DNT) and TNT, except the nitro groups were replaced with NDO substituents [36]. The synthesis began by converting the nitro group of TNT or DNT by reacting with zinc and ammonium chloride in aqueous conditions to yield the intermediate hydroxylamine (scheme 6(ii)). The hydroxylamine was then reacted with manganese dioxide in dichloromethane to yield the intermediate nitroso (scheme 6(iii)). Next, the nitroso is then reacted with t-BuNBr_2_ to yield the tert-butyl-azoxy intermediate (scheme 6(iv)), which is then reacted with NO_2_BF_4_ to yield the final products, **21** and **22** (scheme 6).

**Scheme 7:.**
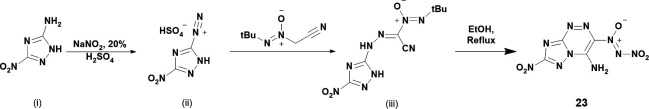
Synthesis of NDO **23** bearing a triazolo-triazene backbone. Scheme is reproduced from a 2019 work by Tartakovsky *et al.* [37].

**Figure 6.**
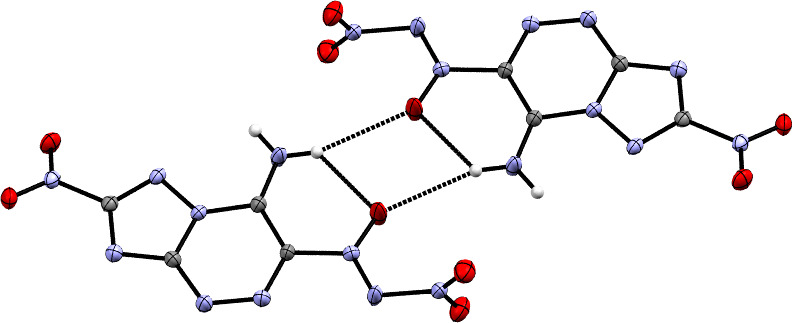
Crystal structure of NDO **23** from a 2019 work by Tartakovsky *et al.* [37].

In Tartakovsky’s 2019 work on annulated NDOs, he reports a NDO on a triazolo-triazine backbone, a new addition to NDO backbones [37]. The synthesis begins with a reaction of 3-amino-5-nitro-1,2,4-triazole with sodium nitrite in 20% sulphuric acid at 0–5°C for 1 hour to yield the intermediate diazonium. To the diazonium, tert-butyl azoxy acetonitrile is added at 0–5°C and stirred for 1 hour, then allowed to warm to room temperature over 12 hours. To convert the hydrazone intermediate into the triazene, refluxing in ethanol was necessary. After the cyclization of the triazene, reaction with six equivalents of NO_2_BF_4_ at –30°C in dry acetonitrile yielded **23**. After the addition of NO_2_BF_4_, the temperature was allowed to rise to 25°C and stirred for a day at this temperature (scheme 7). Perhaps the most notable feature of this NDO is its relatively high thermal decomposition temperature of 154°C, much higher than the usual decomposition temperature for molecules bearing the NDO moiety of 80–120°C. The unusually high decomposition temperature may be attributed to the combination of intra- and inter-molecular hydrogen bonding, evident in the crystal structure (figure 6).

**Scheme 8:.**
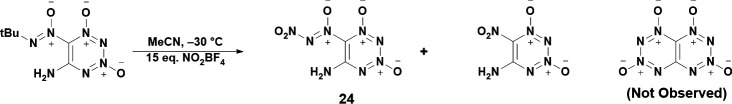
Synthesis of NDO **24**. Scheme is reproduced from a 2019 work by Tartakovsky *et al.* [38].

The same year, Tartakovsky reported the synthesis of a NDO on a 1,2,3,4-tetrazine-1,3-dioxide. Originally, the expected result of reacting the amino t-Bu-azoxy tetrazine dioxide with NO_2_BF_4_ was to yield two tetrazine dioxides on the same C=C backbone; however, the observed products were 5-amino-6-nitro-1,2,3,4-tetrazine-1,3-dioxide and 5-amino-6-(nitro-NNO-azoxy)-1,2,3,4-tetrazine-1,3-dioxide (**24**) instead [38]. To yield **24**, the precursor was reacted with 15 equivalents of NO_2_BF_4_ in dry acetonitrile at –30°C and allowed to stir for 4 days after warming to 25°C (scheme 8).

**Scheme 9:.**
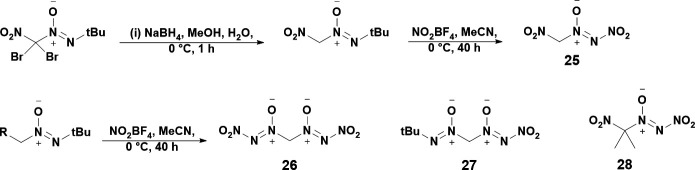
General scheme for the synthesis of NDOs **25–28**. Scheme is reproduced from a 2019 work by Tartakovsky *et al.* [39].

Also in 2019, Tartakovsky reported the first synthesis of aliphatic compounds bearing the NDO moiety [39]. Such work demonstrates that NDOs may be synthesized on not just furazans, tetrazines and aryl groups, but also aliphatic groups. Synthesis of four new NDOs is reported in this paper, **25**, **26**, **27** and **28**, all of which were synthesized by reacting the intermediate tert-butyl-azoxy with NO_2_BF_4_ in acetonitrile at 20°C for 24–42 hours to yield the requisite NDO (scheme 9). Although the main motivation for this work was to study the energetic salts of **25–27** via deprotonating their active methylene protons, these compounds were found to be unstable at room temperature and would decompose spontaneously when dried from organic solvents at ambient conditions. Accordingly, **25–27** were studied predominantly with ^1^H and ^13^C nuclear magnetic resonance (NMR). **28** was the only stable NDO synthesized in this work and was isolated as an oil.

**Figure 7.**
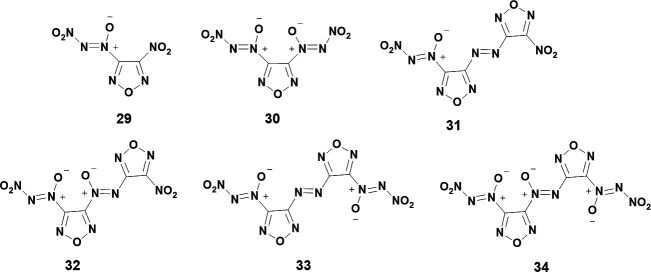
NDOs **29–34** synthesized in a 2020 work by Tartakovsky *et al.* [40].

The last publication to date on the synthesis of new NDOs comes from Tartakovsky and focuses on furazan backbones [40]. Compounds **29–34** are all synthesized in this work (figure 7). Although most followed the usual synthesis pathway as outlined in scheme 3, **30** followed a unique synthetic route. Instead of forming the nitroso to couple with t-BuNBr_2_, the amino-furoxan precursor was immediately able to be coupled with t-BuNBr_2_ [40]. In the article, it is postulated that this immediate coupling was possible as furoxans may have a stable resonance structure with its open-chain counterpart which itself has an exposed nitroso to couple with t-BuNBr_2_. Additionally, Tartakovsky includes a variety of synthetic conditions for the nitration of the tert-butyl-azoxy-furazan intermediate and notes the optimal conditions for most compounds was 35°C with 10 equivalents of NO_2_BF_4_ to yield the NDO in 2–4 hours.

### 2.4. Experimental properties

Some unique properties of NDOs generally are the ONNO bond torsion angles and the N–NO_2_ bond length. In all available crystal structures, the ONNO torsion angle is approximately within the range of 78–88°, which indicates the terminal nitro group is out of plane with the azoxy group in NDOs (table 1). Due to this torsion angle being very close to normal, little sharing of π electrons is possible. Limited sharing of π electrons results in a weak N–NO_2_ bond, and ultimately manifests in the longer than average N–NO_2_ bond length of NDOs relative to the average N–N bond length of 1.47 Å [30]. A summary of bond lengths and angles of select NDOs may be seen in table 1.

**Table 1.** Summary of N–NO_2_ bond lengths and ONNO torsion angles of select NDOs.

compound	N–NO_2_ cond length (Å)	O_2_N–N–N torsion angle (°)
3 [29]	1.48	88.6
7 [29]	1.475	87.8
18 [33]	1.503	84.5
23 [37]	1.464	78.27
Average N–N single bond [30]	1.47	N/A

As a result of the long N–NO_2_ bond length, most NDOs have unique IR stretching frequencies for the N–NO_2_ stretch relative to standard nitro group stretching frequencies (table 2). While the asymmetric NO_2_ stretching mode is relatively unaffected in comparison with an average nitro group, the symmetric stretching frequency is significantly reduced. Relative to an average nitro group, the symmetric stretching frequency is approximately 100 cm^−1^ lower while the asymmetric stretching frequency is approximately 70 cm^−1^ elevated for the NDO. However, stretching frequencies of *n*=N and N(O)=N, the azoxy functional group, are within expected values. Raman spectroscopy was also only reported for **1** and found ν^as^ (NO_2_) of 1612 cm^−1^, ν^s^ (NO_2_) of 1271 cm^−1^, ν (*n*=N) of 1492 cm^−1^ and ν (N(O)=N) of 1319 cm^−1^ in the solid state. Accordingly, IR or Raman spectroscopy may be a useful analytical technique to detect the presence of a NDO by its altered NO_2_ asymmetric and symmetric stretches.

**Table 2.** Summary of select IR data (on KBr) from Tartakovsky *et al.*’s 1996 spectroscopic study of NDOs [27].

compound	*ν* ^as^ (NO_2_) (cm^−1^)	*ν* ^s^ (NO_2_) (cm^−1^)	*ν* (*n*=N) (cm^−1^)	*ν* (N(O)=N) (cm^−1^)
1	1612	1270	1491	1315
2	1600	1256	1501	1316
3	1607	1280	1480	1307
4	1636	1276	1480	1318
5	1610	1286	1496	1318
6	1638	1283	1498	1320
7	1630	1275	1492	1300
8	1627	1284	1496	1330
9	1606	1309	1496	N/A
10	1620	1280	1495	1330
11	1641	1276	1494	1320
12	1626	1266	1478	1306
13	1630	1267	1515	1297
14	1636	1280	1500	1318
15	1632	1280	1509	1315
16	1629	1280	1502	1310
Average ± s.d.	1624 ± 13	1278 ± 11	1495 ± 10	1315 ± 9
Average-NO_2_ [41]	1550–1565	1368–1383		
Average N(O)=N [42]	N/A	N/A	1495–1530	1335–1250

Most NDOs synthesized so far fall within the range of 83–132°C for their decomposition temperature, except for **23**, which decomposes at 154°C. Refer to table 3 for additional reports on NDO decomposition temperatures. **23**’s unusually high decomposition temperature may be explained by intermolecular hydrogen bonding within its crystal structure, something which no other NDO crystal structure possesses to date. Accordingly, NDOs are unlikely to be used for practical energetic materials unless new NDOs with improved decomposition temperatures can be synthesized to be more comparable to RDX at 210°C [3].

**Table 3.** Decomposition temperatures of select NDOs.

Compound	decomposition temperature (°C)
18 [33]	132.1
23 [37]	154
28 [39]	104
29 [40]	109
30 [40]	93
31 [40]	109
32 [40]	115
33 [40]	83
34 [40]	95

Some NDO compounds synthesized have well-known energetic counterparts in the chemical literature which have –NO_2_ groups in place of NDOs. Some of these pairs are **22** with TNT, **23** with 4-amino-3,7-dinitrotriazolo-[5,1 *c*][1,2,4] triazine (DPX-26), **30** with dinitrofurazan (DNF), **33** with dinitroazofurazan (DNAzF) and **34** with dinitroazoxyfurazan (DNAF). Generally, the NDO has a higher enthalpy of formation than its nitro-bearing counterpart, with **22** having the least improvement at 114 kJ mol^−1^ and **34** having the most improvement at 580 kJ mol^−1^. Additionally, NDOs have somewhat higher densities than their nitro-bearing counterparts: **22** is 0.02 g cm^−3^ greater than TNT, **23** is 0.015 g cm^−3^ greater than DPX-26 and **33** has an improvement of 0.13 g cm^−3^ compared with DNAzF. Detonation velocities and detonation pressures are also higher than their nitro counterparts. For example, **22** boasts an improvement in detonation velocity of 399 m s^−1^ and 3.9 GPa for detonation pressure in comparison with TNT. A summary of select NDO performances may be seen in table 4. Despite these improvements to the performance of NDOs relative to nitro groups, the reduced decomposition temperature is a major shortcoming that must be addressed for NDOs to become a viable explosophoric functional groups for real-world application.

**Table 4.** Summary of select NDO energetic properties with comparison with their nitro counterparts.

	22 [36]	23 [37]	30 [40]	33 [40]	34 [40]	TNT [43]	DPX-26 [44]	DNF	DNAzF	DNAF
*ρ* (g cm^−3)^	1.67^a^	1.875^a^	1.70^b^	1.87^a^	1.71^b^	1.65	1.86^a^	1.62^a^, [45]	1.74^a^, [46]	1.82 [47]
*T*_m_ (°C)^c^	114	N/A	Oil	83	Oil	81	N/A	Oil [45]	56 [48]	112 [47]
*T*_d_ (°C)^d^	137	154	93	83	95	309	232	220 [45]	210 [45]	206 [45]
Δ*H*_f_ (kJ mol^−1^)	55.4	655	759^e^	1284^e^	1228^e^	–59	398.3	230^e^ *,*[49]	705^e^ *,*[47]	648^e^ [47]
*ν*_D_ (m s^−1^)^f^	7640	8840	8240	9400	8690	7241	8700	7720, 40]	8710,[40]	8930, [40]
*P*_C-J_ (GPa)^g^	23.9	37.4	29.4	43.4	33.2	21	32	24.0, [40]	33.9, [40]	38.4, [40]

^a^ Density measured by single crystal X-ray diffraction at 298 K.

^b^ Density measured by gas pycnometer.

^c^ Melting temperature measured by DSC.

^d^ Onset of decomposition measured by DSC with ramp rate of 5°C min^-1^.

^e^ Enthalpy of formation calculated by CBS-4M method.

^f^ Calculated detonation velocity.

^g^ Calculated detonation pressure.

## 3. Conclusions

Though NDOs have been in the chemical literature for three decades, their incorporation into energetic backbones remains relatively unexplored in comparison with the nitro group. Where comparisons are available between NDOs and nitro groups, NDOs tend to have superior densities, enthalpies of formation, detonation pressures and detonation velocities than their nitro-bearing counterparts.

However, the decomposition temperature of NDO functional groups has always been lower than that of a nitro group in an equivalent position. Accordingly, the incorporation of known NDOs into energetic formulations is not likely at this time. If an NDO with a decomposition temperature of greater than or equal to 200°C is synthesized, its incorporation into energetic formulations promises to produce formulations with improved density, enthalpy of formation, detonation velocity and detonation pressure relative to its nitro-bearing counterpart. Therefore, further investigation into NDOs on systems which possess intermolecular hydrogen bonding would be candidates for NDOs with improved thermal stability than is currently available. Additionally, it may be possible to improve the thermal stability of NDOs by forming a cocrystal; however, no such work has been attempted to the best of our knowledge at this time.

## Data Availability

This article has no additional data.
